# Quality of life and physical activity levels in kickboxing practitioners: a comparative study

**DOI:** 10.3389/fpsyg.2026.1810181

**Published:** 2026-06-15

**Authors:** Jonatas Deivyson Reis da Silva Duarte, Johan Robalino, Emerson Franchini, Victor Hugo V. Carrijo, Camilla Cavasin Andreato, Ruberlei Godinho de Oliveira, Carlos Alexandre Fett

**Affiliations:** 1 Sports Analysis Laboratory, Federal University of Mato Grosso (UFMT), Cuiabá, Brazil; 2 Postgraduate Program in Health Sciences, Faculty of Medicine, Federal University of Mato Grosso, Cuiabá, Brazil; 3 Porto Biomechanics Laboratory (LABIOMEP), Faculty of Sport, University of Porto, Porto, Portugal; 4 Center for Research, Education, Innovation and Intervention in Sport (CIFI2D), University of Porto, Porto, Portugal; 5 Martial Arts and Combat Sports Research Group, Sport Department, School of Physical Education and Sport, University of São Paulo, São Paulo, Brazil

**Keywords:** combat sports, martial arts, physical health, psychological health, well-being

## Abstract

This study aimed to compare the various domains of quality of life between practitioners and non-practitioners of kickboxing. The study involved 264 volunteers, of whom 153 (90 males and 63 females) were kickboxers and 111 (57 males and 54 females) were sedentary controls. Comparisons were made between black belt (*n* = 63) and colored belt (*n* = 90) and all subgroups with sedentary controls (*n* = 111). To assess physical activity levels, the International Physical Activity Questionnaire was utilized, while quality of life was measured using the World Health Organization Quality of Life Questionnaire. The Mann-Whitney U test was used to compare kickboxers with sedentary controls, with a significance level established of *p* < 0.05. Among kickboxers, 87% (*n* = 133) were classified as having a high level of physical activity, while 13% (*n* = 20) had a moderate level. Conversely, in the sedentary controls group, 86.5% (*n* = 96) were classified as having a low level of physical activity and 13.5% (*n* = 15) as having a moderate level. Kickboxers were significantly older than sedentary controls, with a median of 30 years (IQR: 23–38) compared to 24 years (IQR: 20–31) among sedentary controls (*p* < 0.01), respectively. Black belt kickboxers and competitive athletes were taller than sedentary controls with 1.76 m (IQR: 1.70–1.80) compared to 1.70 m (IQR: 1.60–1.80) (*p* < 0.01). Competitive athletes were also taller than recreational practitioners, with a median height of 1.76 m (IQR: 1.70–1.80) compared to 1.71 m (IQR: 1.60–1.80) (*p* < 0.05). The results of quality of life indicated that kickboxers (recreational, competitive, black and colored belts) had superior quality of life in all domains physical, psychological, social, and environmental when compared to sedentary controls (*p* < 0.001). Competitive kickboxers demonstrated higher physical and psychological domains compared to recreational kickboxers (*p* < 0.05). Nevertheless, black belts only had superior psychological domains than colored belts (*p* < 0.05). Kickboxing appears to be an excellent option for promoting quality of life in the population. Competitors have a partially higher quality of life than recreational athletes, and black belts appear to have better psychological health than colored belts. Therefore, prospective longitudinal studies are warranted to elucidate the causal relationships and underlying effects.

## 1 Introduction

Quality of life is a broad concept that encompasses various areas of a person’s life, and is related to physical and psychological health, positive self-perception of the world, good social relationships, and independence (Cai et al., 2021). It is shaped by objective conditions, such as health, education, and income, and their subjective evaluation, collectively influencing an individual’s perception of well-being (Croes et al., 2018). Additionally, quality of life reflects the perceived sense of physical and mental well-being, underscoring its importance as a focal point in medical, social, and psychological research (Vanleerberghe et al., 2017; Zhang et al., 2016; Shoup et al., 2008). Among the numerous factors influencing quality of life, physical exercise has emerged as a critical determinant, with studies consistently demonstrating that regular exercisers report higher levels of perceived quality of life compared to sedentary individuals (de Oliveira et al., 2019; Yazicioglu et al., 2012).

Physical activity, defined as any systematized bodily movement requiring energy expenditure, is recognized for its extensive health benefits, including the reduction of risks associated with non-communicable diseases such as hypertension, coronary heart disease, stroke, diabetes, certain cancers, and depression (WHO, 2024). The physical exercise can be practiced at low, moderate and high intensity (Albarrati et al., 2018). To optimize health outcomes, the American College of Sports Medicine recommends engaging in at least 150 min of moderate-intensity or 75 min of vigorous-intensity physical activity per week (American College of Sports Medicine [ACSM], 2014). This evidence highlights the pivotal role of regular physical activity in promoting physical and psychological well-being, thus contributing significantly to an improved quality of life. On the other hand, a sedentary lifestyle is associated with chronic non-communicable diseases such as diabetes mellitus, obesity, hypertension, cancer risk, cognitive impairment and depression (Park et al., 2020), increasing the mortality risk of the population that adopts this lifestyle (Chuang et al., 2024).

Combat sports represent a dynamic and multifaceted category, combining technical skill development with physical conditioning (Waşik and Wójcik, 2017). Among the various forms of physical exercise, kickboxing, one of the most globally recognized combat sports, achieved official recognition from the International Olympic Committee in 2021 (IOC, 2021). This striking-based discipline incorporates punches, kicks, and knee techniques within a structured competitive framework (Wako, 2020). Its intermittent and high-intensity nature demands advanced physical attributes such as strength, agility, and endurance, making it a rigorous yet rewarding activity (Slimani et al., 2017). Furthermore, kickboxing’s adaptability allows it to be practiced by diverse populations, ranging from children (Soyib et al., 2024) to adults (Ouergui et al., 2014) and older adults (Lin et al., 2022), highlighting its accessibility and broad appeal as a form of physical activity.

Kickboxing can be practiced for both recreational and competitive purposes, each with distinct training methodologies and objectives (Gümüşay and Koç, 2022). Recreational training emphasizes mastering fundamental skills such as punches, kicks, and knees, alongside health-focused physical conditioning. In contrast, competitive training involves sparring, which refines technical proficiency and hones tactical strategies (Ouergui et al., 2016). Progression within the sport is traditionally marked by a belt-ranking system, where practitioners advance through colored belts, such as white, yellow, orange, green, blue, and brown, and ultimately achieve the black belt, a symbol of mastery and teaching ability (da Silva Duarte et al., 2023; Ritschel, 2008). This progression through the belt-ranking system and the distinct training methods in recreational and competitive kickboxing highlights the potential for varying impacts on overall well-being.

While kickboxing’s health benefits have been explored in specific contexts, such as its effects on the quality of life for individuals with multiple sclerosis (Jackson et al., 2012) and comparisons with alternative practices like yoga (Tsos et al., 2017). Despite existing research on physical conditioning (Ouergui et al., 2014), mood state (da Silva Duarte et al., 2022), and body composition (Eggleton et al., 2018), substantial gaps in the literature persist. Specifically, no studies have examined how the quality of life of kickboxing practitioners compares to sedentary controls, nor have they investigated differences across subgroups, including recreational versus competitive athletes or colored versus black belt practitioners. This could create further evidence of the benefits of kickboxing, which could impact its demand among the adult population and public health policies (social projects based on kickboxing as a strategy to promote population health). Addressing these gaps is crucial for a deeper understanding of kickboxing’s potential role in enhancing overall quality of life. Existing evidence suggests that regular physical exercise is associated with higher quality-of-life scores in children, adults, and older adults (de Oliveira et al., 2019; Schwartz et al., 2021; Moeijes et al., 2019), and it is anticipated that competitive combat sports may report higher quality of life values compared to recreational participants (Kotarska et al., 2019). Our study holds academic and scientific relevance and contributions by enhancing the understanding of how participation in combat sports, such as kickboxing, influences the quality of life and psychological well-being of individuals.

This study aimed to compare the quality of life between kickboxing practitioners and sedentary controls, using the World Health Organization Quality of Life Questionnaire (WHOQOL-BREF) to assess quality of life, and the International Physical Activity Questionnaire (IPAQ) to evaluate physical activity levels of groups. Secondary objectives included comparing the quality of life across subgroups of kickboxing practitioners, specifically by competitive versus recreational status, black belt versus colored belt rank, and male versus female participants. It was hypothesized that kickboxers would report higher quality-of-life scores than sedentary controls. Among kickboxers, competitive athletes were expected to have higher quality of life scores than recreational practitioners, while black belt practitioners were anticipated to report better quality of life than colored belts. Key terms used in this study are defined below (Table 1).

**TABLE 1:** Definitions of the main terms used in the study.

Term	Definition
Kickboxing	Strike combat sport that combines punching, kicking, and kneeing techniques (Niewczas et al., 2024; Rydzik et al., 2024; Wako, 2020; Ritschel, 2008).
Kickboxers	Kickboxing practitioners, including athletes at amateur and professional levels, as well as recreational participants (Ritschel, 2008; Ambroży et al., 2020; da Silva Duarte et al., 2026a).
Competitive training	Structured activities focused on preparing athletes for official competitions (Eime et al., 2013).
Recreational training	Physical and technical activities aimed at health, enjoyment, and skill development, without the primary goal of competition (Eime et al., 2013).
Black belts	Advanced category representing a high level of skill and experience in kickboxing. Indicated by the belt tied around the waist (da Silva Duarte et al., 2023; Ritschel, 2008).
Colored belts	Beginner/intermediate categories indicating gradual skill levels. Represented by belts tied around the waist (Ritschel, 2008).
Sparring	Combat simulation using the equipment and rules of kickboxing or other striking combat sports (Ouergui et al., 2016; da Silva Duarte et al., 2026b).

## 2 Methodology

This study employed a cross-sectional quantitative design with a convenience sampling method. Data collection was conducted online via a Google Forms link (open to everyone) and targeted coaches, athletes, and university students from various Brazilian states. The link was distributed through email, Instagram, and WhatsApp, accompanied by an invitation message prepared by the authors explaining the objectives of the study. Additionally, researchers visited combat sports academies, neighborhoods, and universities to approach individuals in person, provide information about the research, and invite them to participate. It was requested that coaches and athletes who received the questionnaire link (via email, WhatsApp, or Instagram) share it with other colleagues who practice kickboxing. The following instruments were administered through Google Forms: the Informed Consent Form, the International Physical Activity Questionnaire (IPAQ; Craig et al., 2003), the WHOQOL-BREF (Harper et al., 1998), and a brief questionnaire made by the authors, including questions on body mass, height, age, and training duration. Participants were instructed to complete the questionnaires accurately and honestly.

All questions were mandatory, meaning that the system did not allow participants to proceed without answering each item. The questions were derived from validated questionnaires, while the sample characterization items, such as weight, height, age, and duration of practice, were developed by the authors. No artificial intelligence tools were used in the creation of the instruments. Only individuals aged 18 years or older were eligible to participate. Data were collected during the september to november 2024. The identity of the volunteers was kept confidential; names and photos were not disclosed, in accordance with ethical principles. The ethics committee of the Federal University of Mato Grosso - Brazil under number 5.709.528, approved the study.

### 2.1 Sample

The sample size was determined using G*Power software (version 3.1.9.4; University of Düsseldorf, Düsseldorf, Germany). A two-sample *t*-test was conducted to compare the means of two independent groups, assuming a significance level (α) of 0.05 and a statistical power (1-β) of 0.80. Given the absence of prior studies on kickboxing practitioners assessing the domains of the WHOQOL-BREF questionnaire, a moderate effect size (*d* = 0.5) was selected. An allocation ratio of 1 was assumed to ensure an equal number of participants in both the practitioner and non-practitioner groups. The calculation indicated that 64 participants per group were required to achieve sufficient statistical power to detect significant differences across the assessed domains.

A total of 264 volunteers participated in the study, comprising 111 sedentary controls (57 males and 54 females) who served as the control group, and 153 kickboxing practitioners (90 males and 63 females). Among the kickboxers, 75 were classified as competitive athletes and 78 as recreational practitioners. Additionally, 63 participants held black belts, while 90 held colored belts.

The group of kickboxing practitioners included individuals who reported practicing the sport regularly for at least 3 months and who were classified as having a high physical activity level and/or moderate physical activity level on the IPAQ, who train at least 2 times a week. The group of sedentary controls included those who reported not practicing systematic physical exercise for at least 2 months and who were classified as moderate physical activity level and/or low physical activity level by the IPAQ. According to Mujika and Padilla (2000), a period of approximately 1 month without training may already be sufficient to reduce training-induced physiological and performance adaptations.

### 2.2 Instruments

The International Physical Activity Questionnaire (IPAQ)

The IPAQ is a widely used tool for assessing physical activity levels across diverse populations. This questionnaire categorizes physical activity into three levels: high, moderate, and low, based on the frequency, duration, and intensity of activities performed (Craig et al., 2003). Its validity and reliability have been confirmed in the Brazilian context, ensuring its applicability in studies conducted within this population (Pardini et al., 2001).

The World Health Organization Quality of Life (WHOQOL-BREF) questionnaire

The WHOQOL-BREF is a shortened version of the instrument developed by the World Health Organization to assess quality of life. It consists of 26 items distributed across four domains: physical, psychological, social, and environmental. Each item is scored on a 5-point Likert scale, providing a comprehensive measure of quality of life (Fleck et al., 2000; Pedroso et al., 2010).

To ensure data comparability, the raw scores were transformed to a 0–100 scale, following the methodological guidelines of Pedroso et al. (2010). The WHOQOL-BREF has been adapted and validated for use in the Brazilian population, further reinforcing its relevance in local research (de Almeida Fleck, 2000).

The domains evaluated by the WHOQOL-BREF encompass various dimensions of quality of life, as described by Harper et al. (1998):

- Physical domain: Includes aspects such as pain and discomfort, energy and fatigue, sleep and rest, mobility, activities of daily living, dependence on medication or treatments, and work capacity.
- Psychological domain: Covers positive feelings, cognitive processes (thinking, learning, memory, and concentration), self-esteem, body image and appearance, negative emotions, spirituality, religion, and personal beliefs.
- Social domain: Assesses interpersonal relationships, social support, and sexual activity.
- Environmental domain: Examines physical safety and protection, housing conditions, financial resources, the availability and quality of healthcare and social services, recreational and leisure opportunities, and environmental factors such as pollution, noise, traffic, climate, and transportation.

### 2.3 Statistical analysis

All statistical analyses were conducted using SPSS software for Windows (version 29.0.2.0). The Kolmogorov-Smirnov test was applied to assess the normality of the data, which revealed non-normal distributions. Consequently, non-parametric tests were chosen due to their robustness and suitability for data that violate parametric assumptions. Descriptive statistics were reported as medians (Md) and interquartile ranges (first and third quartiles). IPAQ results were expressed as absolute and relative frequencies (%). To compare demographic characteristics, physical activity levels (IPAQ), and quality of life domains between kickboxers and sedentary controls, as well as between male and female kickboxers, the Mann-Whitney U test was employed. Effect sizes (r) were interpreted according to Cohen’s criteria: *r* < 0.10 (small), 0.10 ≤ r < 0.30 (medium), and *r* ≥ 0.50 (large) (Cohen, 1988). For comparisons among more than two groups (e.g., competitive kickboxers, recreational kickboxers, and sedentary controls; black belt kickboxers, colored belt kickboxers, and sedentary controls), the Kruskal-Wallis test was used. When significant differences were identified, Dunn’s *post hoc* test with Bonferroni correction was applied to control for type I error in pairwise comparisons. Effect sizes for the Kruskal-Wallis test were calculated using epsilon squared (ε^2^), with thresholds defined as follows: ε^2^ < 0.01 (small), 0.01 ≤ ε^2^ < 0.06 (medium), and ε^2^ ≥ 0.14 (large). A two-tailed significance level of *p* < 0.05 was adopted for all analyses. The selection of statistical tests and error control procedures was aimed at ensuring methodological rigor, validity, and transparency of the results.

## 3 Results

### 3.1 Comparative analysis of kickboxers and sedentary controls

A comparison between kickboxers (*n* = 153) and sedentary controls (*n* = 111) identified significant differences in anthropometric characteristics, physical activity levels, and quality of life. Kickboxers were significantly older than sedentary controls (*U* = 6361.50, *p* < 0.001, *r* = 0.21). Regarding anthropometric variables, kickboxers exhibited greater height (*U* = 6904.00, *p* = 0.009, *r* = 0.16) and body mass (*U* = 7136.00, *p* = 0.027, *r* = 0.14) compared to sedentary controls, but no significant differences were observed in BMI between groups (*U* = 7801.50, *p* = 0.260, *r* = 0.07).

Physical activity levels, assessed using the IPAQ questionnaire, revealed that 87% of kickboxers were classified as highly active, while none of the sedentary controls reached this level. A moderate activity level was reported by 13% of kickboxers and 13.5% of sedentary controls, whereas 86.5% of sedentary controls were classified as having low activity levels, in contrast to the absence of kickboxers in this category.

Quality of life, evaluated through the WHOQOL-BREF questionnaire, was significantly higher in kickboxers across all domains. In the physical domain, kickboxers scored higher than sedentary controls (*U* = 5272.50, *p* < 0.001, *r* = 0.31). The psychological domain scores were also significantly greater in kickboxers (*U* = 4356.00, *p* < 0.001, *r* = 0.41). Similarly, kickboxers demonstrated superior scores in the social relationships (*U* = 5669.00, *p* < 0.001, *r* = 0.28) and environmental (*U* = 4959.50, *p* < 0.001, *r* = 0.35) domains compared to sedentary controls (Table 2).

**TABLE 2:** General characteristics in median and first and third interquartile values and differences between kickboxers and sedentary controls.

	Kickboxers (*n* = 153)	Competitive kickboxers (*n* = 75)	Recreational kickboxers (*n* = 78)	Black belt kickboxers (*n* = 63)	Colors belts kickboxers (*n* = 90)	Sedentary controls (*n* = 111)
Age (years)	30 (23, 38)*	30 (23, 36)*	30 (24, 40)*	34 (28, 42)*	27 (21, 35)*	24 (20, 31)
Height (meters)	1.73 (1.7, 1.8)	1.76 (1.7, 1.8)*#	1.71 (1.6, 1.8)	1.77 (1.7, 1.8)*	1.72 (1.6, 1.8)	1.70 (1.6, 1.8)
Body mass (kg)	75 (67, 89)§	76 (68, 86)	75 (67, 90)	80 (70, 92)*†	73 (66, 85)	73 (61, 87)
Body mass index (kg/m^2^)	25 (23, 29)	24 (23, 28)	26 (23, 30)	25 (24, 29)	25 (23, 29)	24 (22, 29)
IPAQ (%)						
High physical activity level	133 (87%)	68 (90.7%)	66 (84.6%)	54 (85.7%)	80 (88.9%)	0 (0%)
Moderate physical activity level	20 (13%)	7 (9.3%)	12 (15.4%)	9 (14.3%)	10 (11.1%)	15 (13.5%)
Low physical activity level	0 (0%)	0 (0.0%)	0 (0.0%)	0 (0.0%)	0 (0.0%)	96 (86.5%)
WHOQOL – BREF						
Physical domain	75 (68, 85)*	77 (68, 88)*#	75 (63, 80)*	75 (68, 88)*	75 (63, 80)*	60 (53, 78)
Psychological domain	75 (63, 83)*	77 (65, 88)*#	70 (62, 78)*	77 (65, 88)*†	75 (58, 83)*	62 (48, 70)
Social domain	75 (65, 83)*	75 (65, 83)*	75 (65, 83)*	75 (65, 90)*	75 (65, 83)*	65 (50, 75)
Environment domain	62 (53, 72)*	62 (53, 70)*	62 (53, 75)*	62 (55, 78)*	62 (53, 70)*	52 (43, 63)

WHOQOL than, World Health Organization Quality of Life Questionnaire; IPAQ, International Physical Activity Questionnaire.

*, higher than the sedentary controls values (*p* < 0.001); §, higher than the sedentary controls values (*p* < 0.05); #, higher than the recreational kickboxers values (*p* < 0.05); †, higher than the colors belt kickboxers values (*p* < 0.05).

### 3.2 Competitive kickboxers, recreational kickboxers, and sedentary controls

When the general characteristics of competitive kickboxers (*n* = 75), recreational kickboxers (*n* = 78), and sedentary controls (*n* = 111) were compared, significant differences across multiple variables were found. Age differed significantly among groups (*H* = 12.68, *p* = 0.002), with sedentary controls being younger than both competitive and recreational kickboxers. The effect size was small (ε^2^ = 0.04), indicating a moderate difference.

Regarding height, competitive kickboxers were significantly taller than recreational kickboxers and sedentary controls (*H* = 13.97, *p* < 0.001), with a small effect size (ε^2^ = 0.05). No significant differences were found in body mass among the groups (*H* = 5.32, *p* = 0.069, ε^2^ = 0.02).

Quality of life scores showed a hierarchical pattern, where competitive kickboxers scored highest, followed by recreational kickboxers, with sedentary controls scoring lowest. Notable differences emerged in the physical (*H* = 13.35, *p* < 0.001, ε^2^ = 0.05) and psychological (*H* = 14.17, *p* < 0.001, ε^2^ = 0.06) domains. In the physical domain, significant differences were found between sedentary controls and recreational kickboxers (*p* < 0.001, ε^2^ = 0.91), sedentary controls and competitive kickboxers (*p* < 0.001, ε^2^ = 0.97), and between recreational and competitive kickboxers (*p* = 0.033, ε^2^ = 0.82). The psychological domain showed significant differences between sedentary controls and recreational kickboxers (*p* < 0.001, ε^2^ = 0.94), sedentary controls and competitive kickboxers (*p* < 0.001, ε^2^ = 0.98), and recreational and competitive kickboxers (*p* = 0.006, ε^2^ = 0.89). In social relationships, significant differences were noted between sedentary controls and both recreational (*p* < 0.001, ε^2^ = 0.85) and competitive kickboxers (*p* < 0.001, ε^2^ = 0.91), with no significant differences found between recreational and competitive kickboxers (*p* = 0.507, ε^2^ = 0.02). Finally, in the environmental domain, significant differences were observed between sedentary controls and recreational (*p* < 0.001, ε^2^ = 0.92) and competitive kickboxers (*p* < 0.001, ε^2^ = 0.94), while no significant differences were found between recreational and competitive kickboxers (*p* = 0.753, ε^2^ = 0.00) (Table 2).

### 3.3 Black belt kickboxers, color belt kickboxers, and sedentary controls

The study also compared black belt kickboxers (*n* = 63), colored belt kickboxers (*n* = 90) and sedentary controls (*n* = 111), revealing significant differences in several variables. Age differed significantly between the groups (*H* = 12.16, *p* = 0.002), with black belt athletes being older than color belt athletes (*p* < 0.002, ε^2^ = 0.05), and sedentary controls (*p* < 0.002, ε^2^ = 0.05). The small effect size (ε^2^ = 0.05) indicated a moderate difference in age.

Anthropometric analysis showed that black belt kickboxers were significantly taller than both color belt athletes and sedentary controls (*H* = 7.90, *p* = 0.019, ε^2^ = 0.03). However, body mass (*H* = 5.02, *p* = 0.081, ε^2^ = 0.02) and BMI (*H* = 1.48, *p* = 0.478, ε^2^ = 0.01) did not differ among groups.

Physical activity levels assessed via the IPAQ revealed that 85.71% of black belt and 88.89% of color belt kickboxers were highly active, whereas none of the sedentary controls reached this category. The majority of sedentary controls (70.27%) were classified as having low physical activity levels.

Quality of life scores differed significantly across all WHOQOL-BREF domains. Groups differed in the physical domain (*H* = 28.40, *p* < 0.001, ε^2^ = 0.12). Specifically, sedentary controls differed substantially from colors belt kickboxers (*p* < 0.001, ε^2^ = 0.49) and black belt kickboxers (*p* < 0.001, ε^2^ = 0.58), while colors belt and black belt kickboxers showed no significant difference (*p* = 0.412, ε^2^ = 0.01). A similar result was observed in the psychological domain, (*H* = 50.66, *p* < 0.001, ε^2^ = 0.24). For instance, significant differences were found between sedentary controls and colors belt (*p* < 0.001, ε^2^ = 0.10) and black belt kickboxers (*p* < 0.001, ε^2^ = 0.14), with a lesser effect observed between colors belt and black belt kickboxers (*p* = 0.029, ε^2^ = 0.03). The social relationships domain also differed among groups (*H* = 26.75, *p* < 0.001, ε^2^ = 0.11), with sedentary controls exhibiting significant differences compared to colors belt (*p* = 0.003, ε^2^ = 0.04) and black belt kickboxers (*p* < 0.001, ε^2^ = 0.06). The environmental domain followed the same pattern as the other domains (*H* = 34.45, *p* < 0.001, ε^2^ = 0.15), as significant differences were observed between sedentary controls and colors belt (*p* < 0.001, ε^2^ = 0.04) and black belt kickboxers (*p* < 0.001, ε^2^ = 0.06) (Table 2).

## 4 Discussion

This study was conducted to compare the quality of life of regular kickboxing practitioners with sedentary controls. The main findings of our study indicated that the total sample of kickboxing practitioners, and the subgroups (recreational, competitors, colors, and black belts), had a high quality of life in all domains, compared to sedentary controls, corroborating the hypothesis of this study. When comparing the kickboxing subgroups, it was observed that black belt practitioners only showed high scores in the psychological domain. Competitors, on the other hand, showed higher results in the physical and psychological domains than recreational practitioners. Few studies have thoroughly examined the quality of life of kickboxers, within kickboxing populations remains underdeveloped. This gap highlights the relevance of the present study for the fields of psychology and combat sports, as it contributes to a better understanding of quality of life among kickboxing practitioners.

Our findings are largely consistent with those of Schwartz et al. (2021), as their use of the same quality of life questionnaire, reported significantly higher scores across all quality-of-life domains among practitioners of combat sports and martial arts, such as judo, kung fu, karate, Brazilian jiu-jitsu, and taekwondo, when compared to Brazilian normative values. Similarly, da Costa et al. (2018) observed that physically active older adults exhibited better quality of life than their sedentary counterparts. In Poland, Kotarska et al. (2019) also found that individuals practicing martial arts and combat sports demonstrated higher quality of life and healthier behaviors. Supporting this trend, a cross-sectional study with Portuguese karateka revealed elevated scores in all quality-of-life domains compared to the general population (Tomás et al., 2024). Additionally, a study involving capoeira practitioners showed high scores across all domains relative to Brazilian normative values (Moreira et al., 2022). A recent randomized clinical trial demonstrated that 6 weeks of Muay Thai practice, with 90-minute sessions three times per week, resulted in improvements in participants’ perceived quality of life, love of life, and self-control (Şahin et al., 2025). Collectively, these findings reinforce the growing body of evidence that participation in combat sports, including kickboxing, contributes not only to physical health (physical conditioning, functionality and weight loss) but also to a more positive perception of overall quality of life.

On the other hand, a study by de Oliveira et al. (2019) concluded that older adults who were physically active exhibited a better quality of life compared to sedentary individuals, results that are consistent with those of our study. These findings suggest that regular physical activity is associated with a higher perception of overall well-being. However, studies specifically focused on kickboxing present mixed results. Tsos et al. (2017) using the MOS SF-36 questionnaire concluded that kickboxing practitioners reported a better quality of life than yoga practitioners, while a 5-week intervention study with multiple sclerosis patients using the Multiple Sclerosis Quality of Life Survey instrument did not record improvements in quality of life (Jackson et al., 2012). Our study, being the first to compare the quality of life of kickboxing practitioners with sedentary controls, provides new insights and is pioneering in its use of the WHOQOL-BREF questionnaire in this context. Given that few studies have analyzed the quality of life of practitioners of martial arts and combat sports, this makes further direct comparisons impossible.

In our results, kickboxing practitioners scored higher in the physical domain, corroborating previous findings suggesting that this modality improves various parameters of physical conditioning. Ouergui et al. (2014) demonstrated that 5 weeks of kickboxing training improved both aerobic and anaerobic fitness, flexibility, agility and muscular strength. Other studies have also highlighted the health benefits of kickboxing, such as reductions in blood pressure and body fat percentage (Eggleton et al., 2018), as well as increases in muscle mass and bone density (Lin et al., 2022). Martial arts and combat sports, in general, are recognized for their benefits to physical health (Ambroży et al., 2021; da Silva Duarte and Ferraz, 2022) and for optimizing motor functionality, a key factor for independence and quality of life (Totty and Wade, 2018).

Furthermore, the psychological domain was also higher among kickboxers. Other studies employing different instruments have shown superior psychological health such as, improved mood, resilience, and reduced stress, anxiety, and depression with outcomes in kickboxing practitioners when compared to yoga participants (Tsos et al., 2017) and sedentary controls (da Silva Duarte et al., 2022). These findings support the notion that kickboxing is positively associated with improved psychological well-being. Similarly, research on various martial arts and combat sports has consistently demonstrated beneficial effects on mental health indicators (i.e., improved mood) (Lee et al., 2025; Ciaccioni et al., 2024; Leuzzi et al., 2024; Miyata et al., 2020; Toskovic, 2001).

**FIGURE 1:**
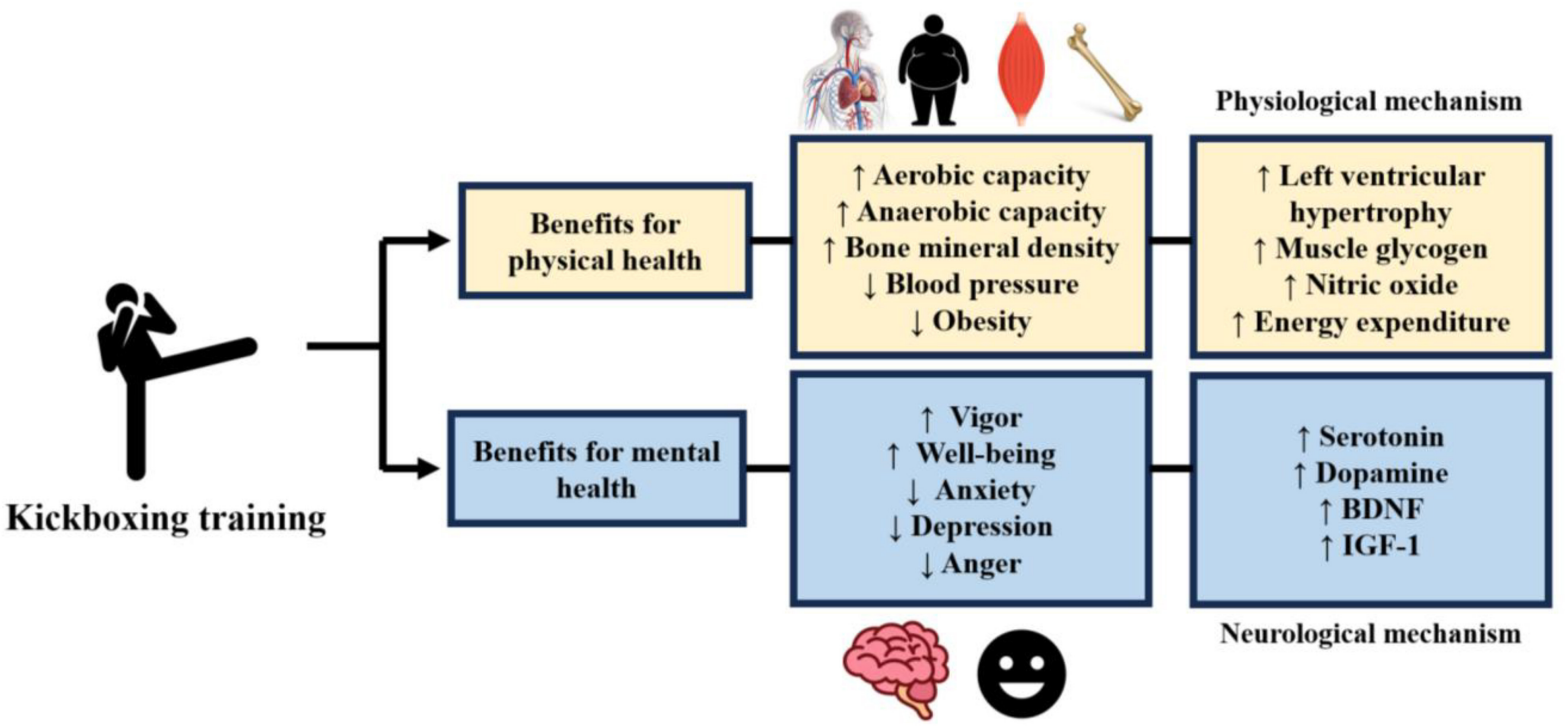
Physical and psychological benefits of kickboxing practice and the underlying physiological and neurological mechanisms. BDNF, brain-derived neurotrophic factor; IGF-1, Insulin-like growth factor 1.

This enhancement in psychological health may be attributed to several physiological and neurochemical factors, including increased serotonin production (Lee et al., 2021; Oh and Kim, 2016) and elevated levels of brain-derived neurotrophic factor (BDNF), a protein linked to mood regulation, memory, neurogenesis and cognitive function in combat sports (Hola et al., 2024; Ozan et al., 2022; Oztasyonar, 2017; Schor et al., 2019; Ziemba et al., 2020). Recent studies have also indicated that the practice of combat sports tends to improve sleep quality (Rosa et al., 2021; Vieira et al., 2021), which may, in turn, positively influence physical recovery and psychological well-being (Charest and Grandner, 2022). Nevertheless, further research is warranted to explore the specific effects of kickboxing on neurotransmitter activity and the underlying mechanisms involved. Additionally, the regular practice of martial arts and combat sports may foster psychological resilience and enhance emotional regulation and BDNF and Insulin-like growth factor 1 (IGF-1) as highlighted by Zou et al. (2018) and Kotarska et al. (2019). Figure 1 illustrates the physical and psychological benefits and their possible physiological and neurological mechanisms.

Regarding the social domain, kickboxers also obtained higher scores. According to Woodward (2009), martial arts and combat sports provide an excellent opportunity for group training. Exercises are often performed in pairs, promoting interaction (Saraiva et al., 2024) and state that martial arts and combat sports are an excellent way to build social connections (Healey et al., 2025). This social aspect is reflected in another study in which kickboxers demonstrated greater social competence compared to practitioners of other disciplines, such as taekwondo and boxing (Çeviker et al., 2021). A 16-week martial arts intervention increased scores on the sociability variable leadership, including group life, being left out, sociability, expressiveness, and patience (Roh et al., 2018). In taekwondo, for example, the social domain was also highlighted as the highest (Shaw and Mukherjee, 2022). These comparisons suggest that, while various combat sports may offer social benefits, kickboxing, like other martial arts, appears to significantly enhance social skills, among them communication and empathy, emphasizing its potential to foster interaction and group cohesion.

In the environmental domain, kickboxers exhibited higher scores, which may be attributed to their regular engagement in health-promoting behaviors, particularly those involving outdoor physical activity (Van Gemert, 2025). However, it is possible that factors other than kickboxing may have influenced this finding, such as access to leisure and healthcare services, transportation, and socioeconomic aspects (Harper et al., 1998). These findings highlight the importance of the environment in quality of life, as a suitable environment positively influences well-being (Interdonato and Greguol, 2010). However, our study included volunteers from different regions of Brazil, which may influence access to kickboxing and other forms of sports and physical exercise. Nevertheless, all participants resided in urban areas, which may have helped to mitigate regional disparities in access.

Comparative analysis between competitive and recreational kickboxing practitioners indicated that competitive athletes achieved significantly higher scores in both the physical and psychological domains. Kotarska et al. (2019) demonstrated that competitive martial artists tend to report higher quality of life scores compared to their recreational counterparts. One potential explanation for this difference could be the more intense training regimen of competitive athletes, who often train multiple times per day (Franchini and Takito, 2014; James et al., 2013). This higher training volume likely contributes to enhanced physical fitness, which is crucial for success in kickboxing competitions (Silva et al., 2016). Furthermore, the intensified training associated with competitive kickboxing may also account for the better psychological outcomes observed in these athletes. Intensive training in competitive kickboxing enhances both physical performance and psychological well-being, guiding strategies to optimize athlete and practitioner development.

Black belts exhibited significantly higher scores in the psychological domain compared to colored belts, likely due to the extensive experience and knowledge acquired over years of practice. Prolonged involvement in combat sports as seen in black belts, has been associated with notable psychological benefits, including increased self-esteem, confidence, and reduced anxiety and stress (Minosky and Dumoulin, 2023). Additionally, this difference may be influenced by age, which showed a statistically significant difference in our comparisons and is linked to greater maturity, emotional regulation, and resilience. Further studies are needed to examine quality of life across belt levels in different combat sports disciplines to better understand the relationship between technical progression, age, and psychological well-being.

The recent study by Tomás et al. (2024) involving karateka found that younger individuals reported higher levels of subjective well-being. This finding contrasts with our results, kickboxing practitioners (older than the sedentary controls group) demonstrated higher quality of life scores. One possible explanation lies in the positive relationship between regular sports participation and happiness, as evidenced by a cross-sectional study (Ruseski et al., 2014). This factor may have contributed to the higher quality of life observed among the older participants in our study. On the other hand, the age difference between the kickboxer and sedentary controls groups was minimal, as reflected by a small effect size (*r* = 0.21).

Some limitations of this study should be noted, including the cross-sectional design, while valuable for capturing a snapshot of the quality of life among kickboxers and sedentary controls, limits our ability to establish causality. With this type of study, it is not known whether kickboxing improves quality of life or whether those who have a good quality of life seek out kickboxing (Schwartz et al., 2021). Furthermore, the findings, though compelling, are inherently tied to the specific population studied and may not fully generalize to other groups or contexts. The potential influences of unmeasured variables such as diet, sleep, or socioeconomic status, geographic state, which could have subtly shaped the results. Additionally, seasonal variations may influence the environmental domain; however, all participants completed the questionnaires during the same period of the year, which may have reduced potential bias related to this aspect.

The reliance on self-reported data, a common challenge in studies of this nature, introduces the possibility of bias to desirabilty bias (da Silva Duarte, 2023). The observed age difference suggests the presence of selection bias in our study, a phenomenon that can occur even in methodologically well-planned research (including randomized studies) (Hernán et al., 2004; Schulz et al., 2010). Moreover, the absence of a longitudinal approach leaves unanswered questions about the sustained effects of kickboxing on quality of life. The cultural context in which this research was conducted may limit its broader applicability. Finally, the level of physical activity may represent a confounding factor; therefore, it is not possible to determine with certainty whether the observed difference is attributable to kickboxing *per se* or to physical activity itself.

## 5 Conclusion

Kickboxers exhibit higher levels of physical activity and superior scores across all domains of quality of life compared to sedentary controls. This benefit is particularly pronounced among competitive kickboxers and those holding black belts, regardless of gender. These findings suggest that intensive and prolonged kickboxing practice may be associated with these positive outcomes, reflecting the influence of training intensity, experience, and mastery of the discipline. However, it is important to recognize that quality of life is a multifactorial construct, and these individuals may have developed such characteristics due to other contributing factors. This underscores the need for future longitudinal studies (clinical trials and cohort studies) to establish a clearer causal relationship.

## Data Availability

The raw data supporting the conclusions of this article will be made available by the authors, without undue reservation.
